# Tree seedling growth allocation of *Castanopsis kawakamii* is determined by seed-relative positions

**DOI:** 10.3389/fpls.2023.1099139

**Published:** 2023-06-02

**Authors:** Jing Zhu, Lan Jiang, Lyuyi Chen, Xing Jin, Cong Xing, Jinfu Liu, Yongchuan Yang, Zhongsheng He

**Affiliations:** ^1^ Key Laboratory of Fujian Universities for Ecology and Resource Statistics, College of Forestry, Fujian Agriculture and Forestry University, Fuzhou, China; ^2^ Entomology and Nematology Department, University of Florida, Gainesville, FL, United States; ^3^ College of Environment and Ecology, Chongqing University, Chongqing, China

**Keywords:** biomass allocation, *Castanopsis* plant, nutrient use efficiency, plant traits, seedling adaptability, trade-off strategy

## Abstract

Plants allocate growth to different organs as a strategy to obtain limiting resources in different environments. Tree seeds that fall from a mother tree settle on, within, or below the forest floor and litter layer, and their relative positions can determine seedling biomass and nutrient allocation and ultimately affect survival to the sapling stage. However, how emerged seedling biomass and nutrients of each organ are affected by seeds in different positions is not yet completely understood in subtropical forests. Therefore, an experiment was conducted with seeds positioned above the litter layers of different thicknesses, on the forest floor, and beneath the litter layer, and the influences of seed position on biomass allocation and nutrient use efficiency of emerged seedlings of *Castanopsis kawakamii* was examined. The aim of the study was to determine the optimal seed position to promote regeneration. Allocation strategies were well coordinated in the emerged seedlings from different seed positions. Seedlings from seeds positioned above litter layers of different thicknesses (~40 and 80 g of litter) allocated growth to leaf tissue at the expense of root tissue (lower root mass fraction) and increased nitrogen (N) and phosphorus (P) accumulation and nutrient use efficiency. Seedlings from seeds positioned beneath a deep litter layer allocated most growth to roots (high root: shoot ratio, root mass fraction) to capture available resources at the expense of leaf growth. Seedlings from seeds positioned on the forest floor allocated most growth to roots to obtain limiting resources. Further, we also found that these traits were clustered into three groups based on trait similarity, and the cumulative interpretation rate was 74.2%. Thus, seed relative positions significantly affected seedling growth by altering the allocation of resources to different organs. The different strategies indicated that root N:P ratios (entropy weight vector was 0.078) and P nutrient use efficiency were essential factors affecting seedling growth in the subtropical forest. Of the seed positions analyzed, beneath a moderate litter layer (~40 g of litter) was the most suitable position for the growth and survival of *Castanopsis* seedlings. In future studies, field and lab experiments will be combined to reveal the mechanisms underlying forest regeneration.

## 1 Introduction

Plants achieve rapid growth by altering allocation strategies to capture the most available resources (i.e., water, light, and nutrients). For example, plants allocate growth to shoots or roots to, respectively, capture light or soil water and nutrients, which indirectly affects plant regeneration (Umaña et al., 2020). In forest ecosystems, seedlings are the most vulnerable stage in plant life histories (Yang et al., 2015), and the early developmental stages of seedlings are more sensitive to environmental alterations than adult stages (Wellstein, 2015). Further, forest community dynamics are unpredictable in subtropical forests due to the complex interaction among tree seedling growth, traits allocation of each organ, and environmental conditions (Bhadouria et al., 2017). Thus, understanding how seedling allocation responds to variations in the environment is a major goal in forest regeneration.

Litter or soil is the initial physical environment that seeds contact after falling from a tree (Ruprecht and Szabó, 2012; Werden et al., 2020), and seeds can settle on, within, or beneath the forest floor and litter layer (Wellstein, 2015). Seeds settle on the forest floor by animal dispersal or gravity (Huang et al., 2021; Perea et al., 2021), and then experience strong but effective photosynthetic radiation (high-intensity light). In this case, emerged seedlings prioritize the investment of resources into roots to expand root area to absorb and store water (water limitation theory) (Ma et al., 2021), thereby altering above- and belowground allocation patterns of seedlings (Umaña et al., 2021). Seeds also settle on top of the litter layer because the physical barrier of litter prevents seeds from reaching the soil (Rotundo and Aguiar, 2005; Nuñez et al., 2021). In this case, the roots of emerged seedlings cannot contact the soil, which affects seedling establishment (Rotundo and Aguiar, 2005). When litter input occurs after seed rain, seeds settle beneath the litter layer. The physical barrier caused by litter must be penetrated for seedling emergence, which alters resource allocation (Donath and Eckstein, 2011). A forest litter layer can reduce light intensity and quality, and regulate the physical and chemical environmental factors, e.g., soil moisture and nutrients, leading to less active heat exchange between the forest floor and canopy (Ruprecht et al., 2010). In this case, litter facilitates seedling growth by providing rich nutrient resources [e.g., nitrogen (N) and phosphorus (P)] (Quested and Eriksson, 2006), and provides important protection and buffering effects for early plant regeneration (Zhang et al., 2022). However, litter also restricts seedling establishment (Zhang et al., 2022), and deep litter can act as a mechanical barrier that causes the extinction of light, which affects seedling emergence (Loydi et al., 2015). Ellsworth et al. (2004) also observed that photosynthetically active radiation reaching the forest floor decreases under deep litter layer. Therefore, under the deep litter, the litter layer negatively affects seedling settlement (Zhu et al., 2022b). Thus, understanding how seed relative positions in relation to the litter layer and forest floor affect the trade-offs between above- and belowground allocation of emerged seedlings will help to clarify plant resource acquisition strategies.

*Castanopsis* is a common tree genus in subtropical evergreen broad-leaved forests and is widely used in forest restoration (Du et al., 2007; Yang et al., 2015). The regeneration dynamics of *Castanopsis* plants directly affect future species composition and community structure of subtropical forest ecosystems (Huang et al., 2020). In this study, we focus on seedlings since it represents an important demographic bottleneck for forest communities, and whether seedlings survive determines the success of natural regeneration (Walck et al., 2011; Yang et al., 2015; Umaña et al., 2016). The *Castanopsis kawakamii* Nature Reserve in Sanming City includes the typical evergreen broad-leaved tree species in subtropical forests throughout southeastern China (He et al., 2019). The canopy is closed because of the high forest biodiversity. There is a deep litter layer in the understory, and seed relative positions are the most common factor affecting seedling recruitment and turnover rate from seedlings to saplings (He et al., 2020). However, the effects of seed relative positions in the litter layer on emerged seedling growth remain unclear. Because of the effects of seed relative positions, regeneration dynamics and recovery strategies of seedlings in the understory are typically uncertain (He et al., 2019; He et al., 2020; Zhu et al., 2022b), presenting a challenge to forest management.

**Figure 1:**
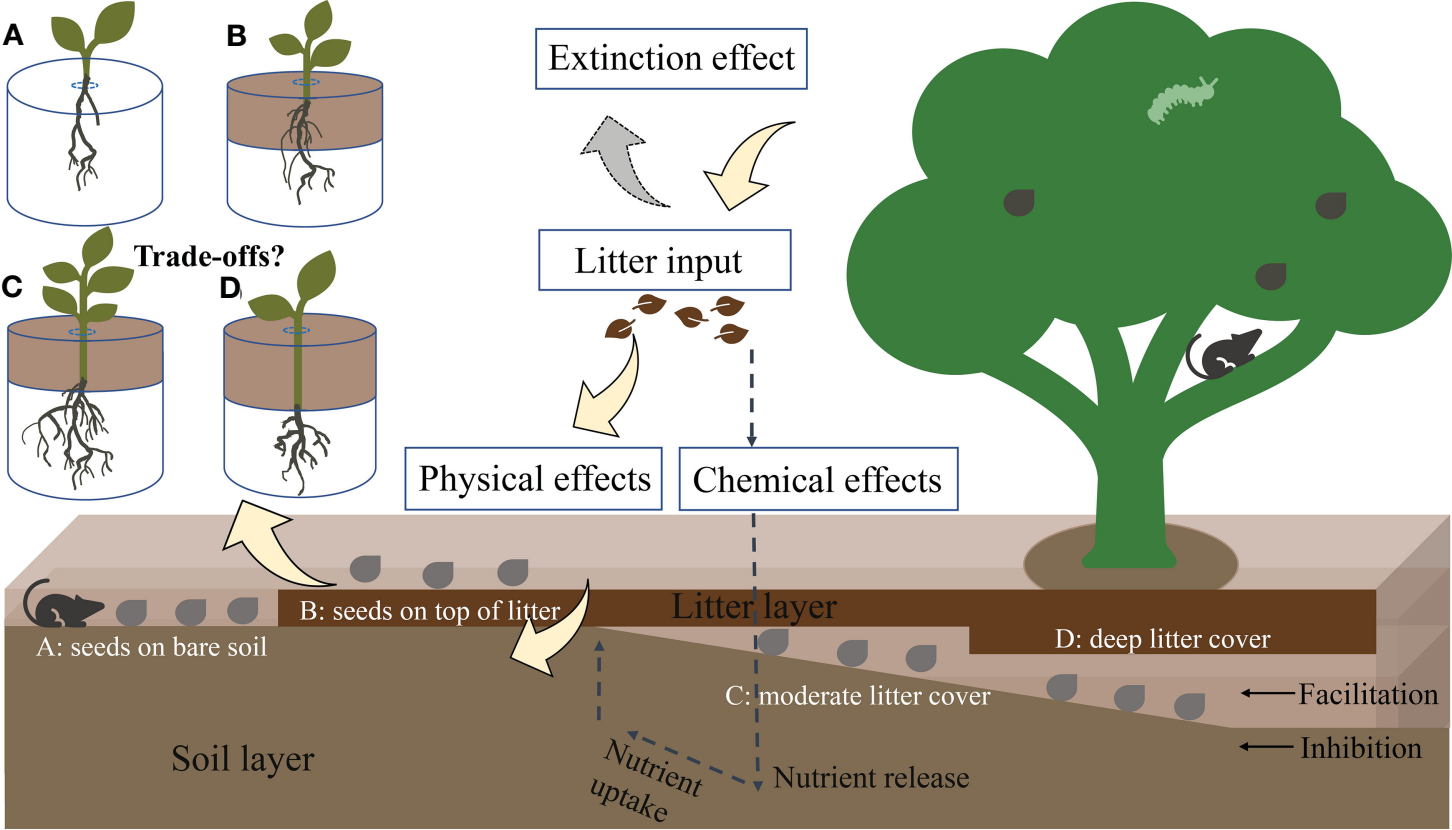
Conceptual model of the physical effects of litter, which cause seed positions to facilitate or inhibit plant regeneration. Yellow, gray, and dashed arrows represent the physical effects of litter, light extinction effects, and possible chemical effects/feedbacks of litter, respectively. Gray points represent seeds fallen from mother trees. **(A–D)** describe different positions of seeds that fall from mother trees. After germinating, seedlings from different seed positions have different growth patterns because of litter thickness and associated physical effects. Experimental design: **(A)** seeds positioned on the forest floor (without litter), **(B)** seeds positioned on top of litter, **(C)** seeds positioned beneath moderate litter cover (2 and 4 cm), and **(D)** seeds positioned beneath the deep litter cover (6 and 8 cm).

Thus, *Castanopsis kawakamii*, one of the most widely distributed dominant tree species in natural evergreen broad-leaved subtropical forest communities, was selected as the research object in this study. This study examined whether differences in allocation to seedling shoots and roots resulting from seed relative positions affected natural regeneration in subtropical forests (**Figure 1**). To understand the relationship between seedling allocation and regeneration, it is essential to determine how seed position in relation to the forest floor and litter layer alters allocation to seedling shoots and roots. Determining how seed position affects seedling resource allocation can help to identify the most suitable seed position for seedling regeneration in a subtropical forest. Therefore, seedling above- and belowground biomass and nutrient content traits were measured to reveal how seed positions affect seedling resource allocation.

## 2 Materials and methods

### 2.1 Site description

The study site was located in the *Castanopsis kawakamii* Nature Reserve in Sanming City, Fujian Province, China (26°07′–26°12′N, 117°24′–117°29′E). The region has a subtropical monsoon climate (He et al., 2019; He et al., 2020). The average annual temperature is 19.5°C, and the average annual precipitation is 1,500 mm. Canopy closure is approximately 80% across the region (He et al., 2019). Vegetation is typical subtropical evergreen broad-leaved forests, with the tree layer co-dominated by *C. kawakamii* (average age > 100 years), *Litsea subcoriacea*, and *Schima superba* (He et al., 2020; Zhu et al., 2022a).

### 2.2 Collection of materials

In March 2018, 10 sampling plots dominated by *C. kawakamii* were established in the *Castanopsis kawakamii* Nature Reserve. Five 1 m × 1 m litter traps were placed 1 m above the forest floor in each plot (Zhu et al., 2022b). To reduce the experimental error caused by differences in litter types, litter from the traps was collected once a month, mixed in the laboratory, and then oven-dried to a constant weight at 65°C for 72 h.

From October to December 2018 (depending on the peak seed fall period), full and intact seeds were collected and then stored in sand. Before conducting a germination experiment, we placed seeds in distilled water for 3 h, removed the floating and inferior seeds, and selected the intact and pest-free seeds for disinfection using 0.5% KMnO_4_. For specific details, see Zhu et al. (2022a).

The relatively thick humus layer (0–10 cm) and intricate plant roots systems on the forest floor of the *C. kawakamii* natural forest prevented seeds from always contacting the forest floor. Thus, soil was collected from a depth of 10–40 cm in each sample plot. The soil from each plot was a mixture of soil from the east, south, west, north, and center positions of each plot. The soil was passed through a 5-mm sieve to remove roots and stones. The soil was then sterilized in an autoclave for 30 min at 120°C to reduce the probability of seeds in the soil being infected by pathogens (Liang et al., 2019).

### 2.3 Experimental design

A germination experiment was conducted in a large indoor laboratory in January 2019. Germination pots measured 38.5 cm × 27.5 cm × 14 cm, and litter weight was calculated according to pot volume. A completely randomized experimental design included seven seed-position treatments. Seeds positioned above the litter layers included one positioned in a 2 cm thick litter layer (~40 g of litter) and one positioned in a 4 cm thick litter layer (~80 g of litter). That is, seeds were in direct contact with the litter layer. Seeds’ relative positions on the forest floor or beneath the litter layer meant direct contact between the seeds and forest soil. Seeds positioned on the forest floor without litter cover layer. Seeds positioned beneath the litter layer meant that they are covered by 2 cm (~40 g of litter), 4 cm (~80 g of litter), 6 cm (~120 g of litter), and 8 cm (~160 g of litter) of litter (Zhu et al., 2022b). Each position included three experimental pots, and each pot was sown with 50 seeds of uniform size (2.062 ± 0.06 g). Thus, the experiment included a total of 21 experimental pots. Litter thickness was determined by weight, and the thickness of litter at each position was kept as consistent as possible during the study. Seeds began to germinate in mid-February, and germination was complete in mid-April 2019. Emerged seedlings, i.e., those that successfully penetrated through the litter cover, were counted every three days until the number of emerged seedlings did not increase on three consecutive days (Wellstein, 2015). Seedlings were cultivated for 6 months after emergence to measure above- and belowground traits. Pots received the same amount of water (200 mL/pot) every two days and were moved regularly to ensure similar environmental conditions for all pots during seedling growth (Zhu et al., 2022b). Soil moisture was determined based on the average annual humidity of the *C. kawakamii* natural forest (18%–27%) (Wang et al., 2021b), and litter thickness reached the mean annual value of litter in the natural forest of *C. kawakamii* (432.5–922.5 g/m^2^).

### 2.4 Seedling biomass

In December 2019, functional traits were measured on individual seedlings. Three surviving individual seedlings in each experimental pot were harvested to measure biomass and nutrients. Fully expanded leaves of seedlings without obvious pests and diseases were selected, and roots were separated from the main stems and leaves. Each organ was carefully cleaned of soil with deionized water, ensuring the integrity of roots as much as possible. Plants’ tissues were placed in marked envelopes and returned to the laboratory for further analysis. Roots, stems, and leaves were oven-dried to constant weight at 65°C for 72 h, and dry matter mass (0.0001 g) was determined to calculate traits related to biomass allocation. Root: shoot ratio was calculated as root biomass divided by the sum of stem and leaf biomass. Mass partitioning into photosynthetic and non-photosynthetic tissues was calculated as photosynthetic tissue (leaf biomass) divided by non-photosynthetic tissue (sum of stem and root biomass) (Umaña et al., 2020). Total plant biomass was the sum of root, stem, and leaf biomass. Root mass fraction, stem mass fraction, and leaf mass fraction were calculated as root, stem, and leaf biomass divided by the total plant biomass, respectively (Shen et al., 2019b). The mean value was determined from the three surviving individual seedlings in each experimental pot (*n* = 3) and was regarded as one available value.

### 2.5 Nutrient contents

Dried seedling roots, stems, and leaves were crushed by mortar and sieved through a 0.15-mm mesh. Total N contents of roots, stems, and leaves were determined by an elemental analyzer (VARIO MAX CUBE, Elementar Analysensysteme GmbH, Germany), and total P contents were determined by inductively coupled plasma emission spectrometer (ICP-OES, PE OPTIMA 8000, PerkinElmer Inc., USA). The N:P ratios in root, stem, and leaf were calculated as N contents divided by P contents of the different tissues. The total N and P contents of plants were determined by biomass-weighted mean values of N and P contents of leaves, stems, and roots. Total N and P accumulation were determined by multiplying total plant biomass and total nutrient contents. Plant nutrient use efficiency was calculated as total plant biomass divided by total plant nutrient content (Bridgham and Pastor, 1995).

### 2.6 Statistical analyses

To analyze differences in biomass and nutrient allocation of emerged seedlings from different seed positions, the normality of all variables was first tested using the “shapiro.test” function. Then, “aov” and “duncan.test” functions in the “agricolae” package were used to identify significant differences among means of seedling traits from different seed positions (de Mendiburu, 2009). To assess relationships within data, effect size and 95% confidence interval (CI) were also tested for significant differences, and ‘effect size’ was replaced with ‘mean difference’ to estimate the magnitude of an effect (Nakagawa and Cuthill, 2007). Pearson correlation analysis was performed using the “corrplot” package (Friendly, 2002) to examine correlations between biomass and nutrient contents of different organs.

To consider the similarity between individuals and reduce the dimension of a primary matrix containing mixed types of variables in each organ of *C. kawakamii* seedlings, biomass and nutrient contents were incorporated into a principal component analysis (PCA) using the “prcomp” function from the “factoextra” package, and the first two dimensions were extracted (Kassambara and Mundt, 2017). The analysis was also used to identify the adaptive strategies of seedlings from seeds in different positions. Furthermore, k-means cluster analysis was used to group similar traits in the “cluster” package based on the nearest neighbor method, and the “factoextra” package was used to determine the optimal number of clusters (Kassambara and Mundt, 2017). The number of points of categorical variables was also extracted, and figures were prepared using the “prcomp” function to classify differences among seed positions. The primary matrix included 19 quantitative variables (including mass partitioning into photosynthetic and non-photosynthetic tissues, root: shoot ratio, total biomass, root mass fraction, stem mass fraction, leaf mass fraction, N and P contents of each organ, N:P ratios of root, stem, and leaf, N accumulation, P accumulation, N nutrient use efficiency, and P nutrient use efficiency) for the seven seed positions. The software R 3.5.3 was used for statistical analyses and the preparation of figures (R Development Core Team, 2020).

To determine the most suitable seed position for *C. kawakamii* seedling growth, the maximum difference normalization method was used to eliminate the effects of different trait dimensions (Zou et al., 2006). The entropy method was used to determine the objective weight of the abovementioned traits in fuzzy synthetic evaluation (Zou et al., 2006). Positive indices were those with high trait values indicating low-stress levels. However, the allocation of biomass and nutrient contents of roots, stems, and leaves represented plant adaptation strategies and explained the degree of environmental stress. Increases in values of different traits indicate plant priorities in obtaining resources. Thus, in this study, all traits were considered positive indicators, and in a synthesis of all the influencing factors, trait entropy weights were calculated. With this approach, comprehensive evaluation scores of different seed positions were obtained (Zou et al., 2006; Zhong et al., 2021), and the most suitable seed position for seedling growth was determined.

## 3 Results

### 3.1 Biomass allocation to roots, stems, and leaves

**Figure 2:**
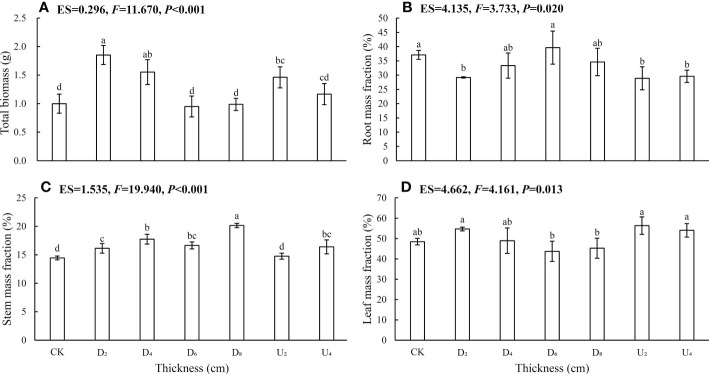
Seedling **(A)** total plant biomass, **(B)** root mass fraction, **(C)** stem mass fraction, and **(D)** leaf mass fraction. CK represents emerged seedlings from seeds positioned on the forest floor (bare soil); D_2_, D_4_, D_6_, and D_8_ represent emerged seedlings from seeds positioned beneath the litter layer with a cover of 2 cm, 4 cm, 6 cm, and 8 cm of litter, respectively; U_2_ and U_4_ represent emerged seedlings from seeds positioned above litter that is 2 cm and 4 cm thick, respectively. Different lowercase letters (a, b) indicate significant differences (P < 0.05). ES indicates the effect size; P indicates the probability value; F indicates the F-test value; and error bars represent one standard deviation, with the same below, and df = 6.

Plant biomass allocation in *C. kawakamii* seedlings from different seed positions was significantly different (**Figures 2**, **S1; Table S1).** Total biomass (**Figure 2A**), root mass fraction (**Figure 2B**), stem mass fraction (**Figure 2C**), and root: shoot ratio (**Figure S1**) of seedlings from seed positions above the litter layer were lower than those of seedlings from seed positions beneath litter layer, with leaf mass fraction being the exception (**Figure 2D**). Biomass values varied significantly with increasing thickness of the litter layer, and root mass fraction (**Figure 2B**), stem mass fraction (**Figure 2C**), and root: shoot ratio (**Figure S1**) of seedlings from seeds positioned beneath the deep litter cover were higher than those of seedlings from other seed positions.

### 3.2 Nitrogen and phosphorus contents in roots, stems, and leaves

**Figure 3:**
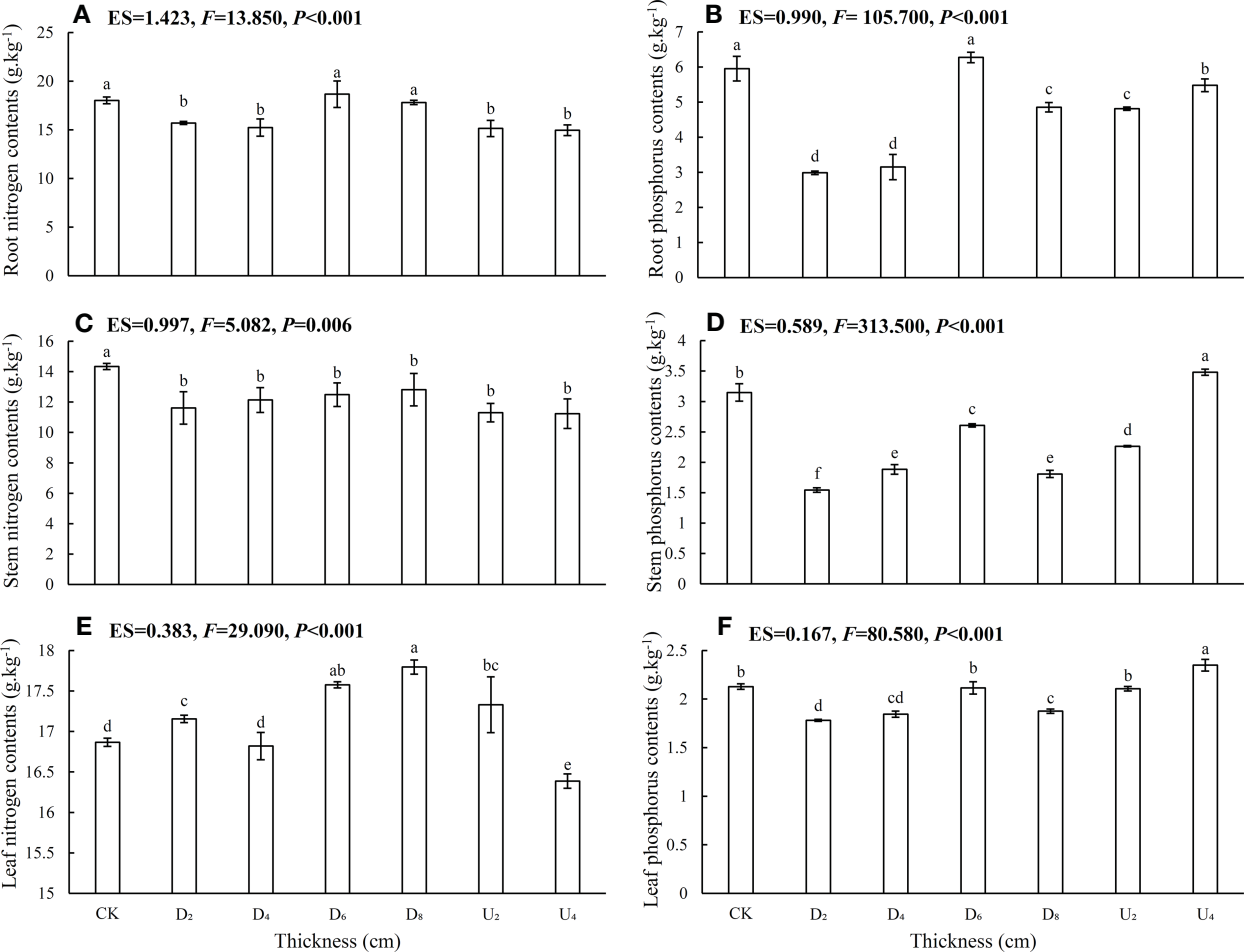
Nitrogen and phosphorus contents in roots, stems, and leaves of *Castanopsis kawakamii* seedlings. **(A)** Root nitrogen contents, **(B)** root phosphorus contents, **(C)** stem nitrogen contents, **(D)** stem phosphorus contents, **(E)** leaf nitrogen contents, and **(F)** leaf phosphorus contents of seedling. Different lowercase letters (a, b) indicate significant differences (P<0.05).

**Figure 4:**
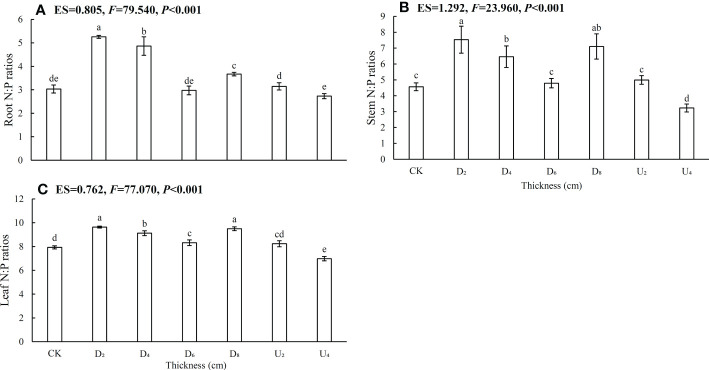
Nitrogen, phosphorus ratios in **(A)** roots, **(B)** stems, and **(C)** and leaves of *Castanopsis kawakamii* seedlings. Different lowercase letters (a, b) indicate significant differences (P<0.05).

In different seed positions, leaf N contents and N:P ratios were overall higher than those in root and stem tissues (**Figures 3**, **4**; **Table S1**), whereas P contents were lowest in leaves, intermediate in stems, and highest in roots. In addition, N contents of roots, stems, and leaves of seedlings from seeds positioned under the deep litter layer and on the forest floor were overall higher than those in seedlings from other seed positions (**Figures 3A, C, E**). Furthermore, P contents of root, stem, and leaf tissues under moderate amounts of litter layer (~40 and 80 g of litter) were overall lower than those in seedlings from other seed positions (**Figures 3B, D, F**), whereas N:P ratios were not (**Figures 4A–C**). Phosphorus contents of the shoot and root tissues of seedlings from seeds positioned above the litter layer were higher than those of seedlings from seeds positioned beneath the litter layer.

### 3.3 Nutrient accumulation and nutrient use efficiency

**Figure 5:**
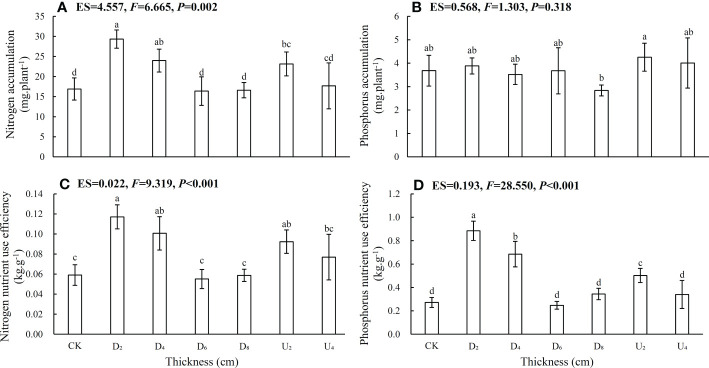
**(A)** Nitrogen accumulation, **(B)** phosphorus accumulation, **(C)** nitrogen nutrient use efficiency, and **(D)** phosphorus nutrient use efficiency of *Castanopsis kawakamii* seedlings. Different lowercase letters (a, b) indicate significant differences (P<0.05).

Seed positions altered whole-plant N and P accumulation and nutrient use efficiency (**Figure 5**; **Table S1**). N accumulation, N and P nutrient use efficiencies of seedlings from seeds positioned under moderate litter layers (~40 and 80 g of litter) were overall higher than those of seedlings from other seed positions, whereas seedling P accumulation was lower than that from seeds positioned above the litter layer. Nutrient accumulation and use efficiency of emerged seedlings from seeds positioned beneath the deep litter layer and on the forest floor were lower than those of seedlings from seeds in other positions.

### 3.4 Correlations between biomass and nutrient contents

**Figure 6:**
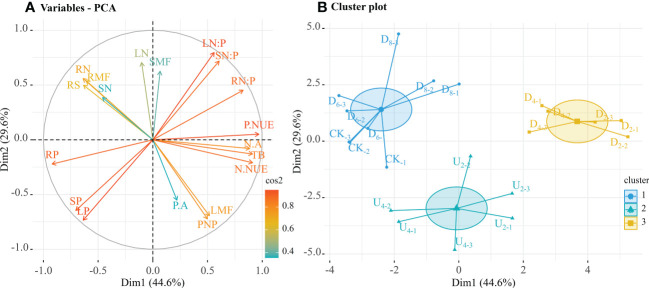
Relationship between biomass and nutrient contents in each organ of seedlings from seeds in different positions. **(A)** First two dimensions of principal component analysis. **(B)** Cluster analysis groups of individual categorical variables (seven seed positions). Cos2 value indicates the contribution of principal components, and cluster indicates the optimal number of clusters. The endpoint of cluster analysis was a set of clusters, with each cluster distinct from the others and objects within each cluster broadly similar to one another. Mass partitioning into photosynthetic and non-photosynthetic tissues (PNP), root/shoot ratio (RS), total plant biomass (TB), root mass fraction (RMF), stem mass fraction (SMF), leaf mass fraction (LMF). Root nitrogen contents (RN), stem N contents (SN), leaf N contents (LN), root phosphorus contents (RP), stem P contents (SP), and leaf P contents (LP). N:P ratios of roots (RN:P), N:P ratios of stems (SN:P), N:P ratios of leaves (LN:P); N accumulation (N.A), P accumulation (P.A), N nutrient use efficiency (N.NUE), and P nutrient use efficiency (P.NUE).

Biomass allocation to each organ was highly significantly correlated with nutrient contents (**Figure S2**). In a PCA, the first two dimensions explained 74.2% of the variability (**Figure 6A**). The first dimension (Dim 1) of the PCA accounted for 44.6% of the variance and indicated relatively high P nutrient use efficiency, root N:P ratios, N nutrient use efficiency, total biomass, and N accumulation, and relatively low root P contents were positively correlated (**Figure S3A**). The second dimension (Dim 2) captured 29.6% of the variance and indicated relatively low leaf N:P ratios, stem N:P ratios, and leaf N contents were negatively correlated (**Figure S3B**).

The nearest neighbor method was used to cluster seedlings from different seed positions according to close and distant relations of trait similarity, and seedlings from seed positions with the greatest trait similarity were clustered together (**Figure 6B**). Principal components were extracted from biomass and nutrient indices, which were then clustered into three groups. One group included emerged seedlings from seeds positioned at different depths beneath the moderate litter layer (~40 and 80 g of litter), which had higher total biomass, nutrient accumulation, and nutrient use efficiency than seedlings in the other groups. A second group included seedlings from seeds positioned on the forest floor and beneath the deep litter layer, which had relatively high root biomass and nutrient contents. The third group included seedlings from seeds positioned on top of the litter layer, which all had high trait similarity.

### 3.5 A comprehensive evaluation of different seed position*s*

**Figure 7:**
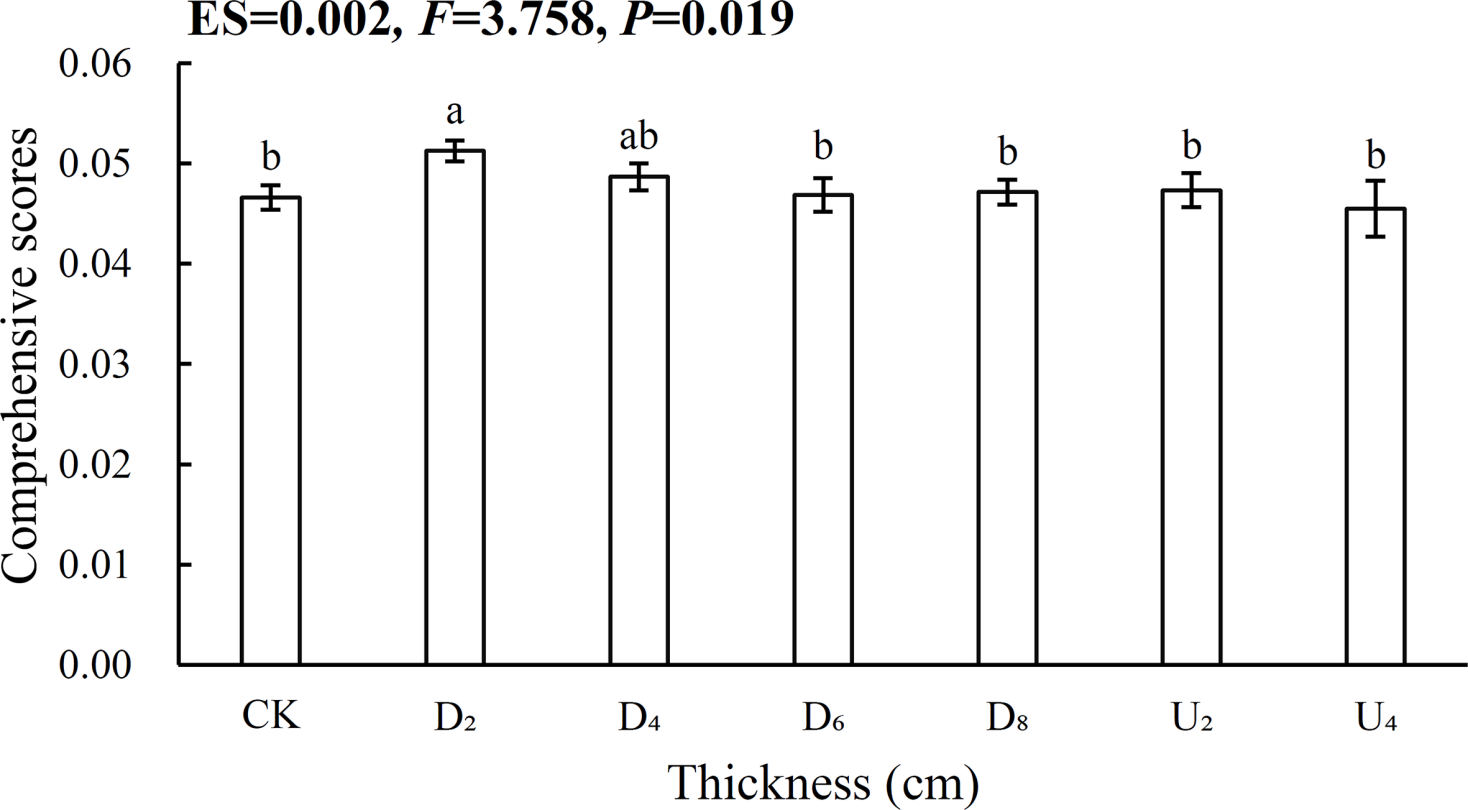
Comprehensive evaluation based on the entropy method for different seed positions. Different lowercase letters (a, b) indicate significant differences (P<0.05).

The entropy weight vectors of biomass and nutrient contents of emerged seedlings indicated that root N:P ratios had the highest entropy weight vector, whereas root mass fraction had the lowest entropy weight vector (**Table S2**). The comprehensive score of the seed position beneath moderate litter cover (~40 g of litter) was significantly higher than that for the other positions, indicating the position had the most significant positive effect on seedling growth (**Figure 7**; **Table S1**). However, the lowest comprehensive score was for the seed position above the litter layer (~80 g of litter), indicating that the position might inhibit seedling growth.

## 4 Discussion

### 4.1 Effects of seed position on trade-off strategies in seedling biomass allocation

In this study, the trade-offs involved in the above- and belowground biomass allocation strategies of seedlings were examined in response to seeds positioned on, within, and beneath the litter layer. Seedlings allocate growth to different tissues to increase the capture of the most limiting resource (Hodáňová, 1981), which ultimately affects the performance of individuals from different seed positions (Weemstra et al., 2021). Biomass allocation patterns indicated that seedlings from seeds positioned beneath the litter layer (~40 and 80 g of litter) had relatively high photosynthetic tissues at the expense of root tissues, whereas seedlings from seeds beneath the deep litter layer (~120 and 160 g of litter) decreased aboveground tissues by facilitating biomass allocation to belowground tissue. Therefore, when one organ receives additional resources, a decrease in investment in another organ is inevitable (Luo et al., 2020), indicating that plants take advantage of trait plasticity to maintain total growth. Litter acts as a physical barrier to seedling establishment (Wellstein, 2015), and the deep litter layer might cause respiration in biological mechanisms that cannot meet the needs for seedling photosynthesis (Zhang and Xi, 2021). To occupy a favorable position in the vertical direction, emerged seedlings from seeds positioned beneath the deep litter layer allocate additional carbohydrates to belowground tissue, and transfer excess nutrients to aboveground tissues to improve the acquisition of resources (Shen et al., 2019a; Ottaviani et al., 2020). Litter directly affects the light intensity reaching the forest floor, and indirectly leads to wilting or plant death (Sayer et al., 2006; Xia et al., 2019). Thus, when seedlings emerge from a seed position beneath a deep litter layer, the resulting variability in light intensity causes shifts in the ecological strategies that increase growth (Umaña et al., 2021). Under a deep litter layer, emerged seedlings increase investments in root biomass at the expense of aboveground tissues to acquire the most available resources and optimize total growth (Hodáňová, 1981). In addition, the presence of a grass layer also plays a major role in tree seedlings’ growth and establishment and competes with the roots of emerged seedlings for available soil resources, thus limiting the rapid seedling growth in this position (Bhadouria et al., 2020). However, litter can also block heat exchange between the soil and the external environment, prevent seedlings from being burned by high light intensities (Donath and Eckstein, 2011), release nutrients by decomposing, as well as reduce the possibility of seed predation (Wellstein, 2015). These interactive effects of light, nutrient availability caused by the litter layer, and the variations of relative positions contribute to seedling survival and recruitment (Bhadouria et al., 2020). When seedlings emerge from seeds positioned beneath or above a moderate litter layer (~40 g of litter), suitable environmental conditions can promote leaf carbon uptake and assimilation and thus facilitate leaf biomass accumulation to increase photosynthesis (de Groot et al., 2003). Simultaneously, plants can convert products of photosynthesis into resources that can be absorbed and used in natural regeneration (de Groot et al., 2003). Thus, in the case of seeds positioned beneath or above a moderate litter layer, the emerged seedlings altered the investment of each organ to receive the most available resources. 

The biomass allocation strategy (i.e., relatively high root biomass) of seedlings from seeds positioned on the forest floor (i.e., bare soil) is consistent with the water limitation theory (Ma et al., 2021). When suffering from water limitation, plants always allocate more energy to belowground tissue to absorb and store water resources, and reduce leaf biomass to decrease water loss caused by transpiration (Ledo et al., 2018; Ma et al., 2021). In this study, emerged seedlings from seeds positioned on the forest floor invested in root biomass at the expense of leaf and stem tissues. The response was likely because rapid water evaporation easily transforms the forest floor into an arid environment, resulting in the lethal desiccation of seedlings (Wellstein, 2015). Furthermore, compared with seedlings germinating in the litter layer, emerged seedlings on the forest floor may be exposed to strong light intensity and experience burnt leaves (Ellsworth et al., 2004). In this position, emerged seedlings might increase allocation to roots to increase access to available resources and continue growth (Boonman et al., 2020). Thus, water balance and light intensity are critical factors in modulating the response of each organ to resource variation to maximize performance. In summary, to capture available resources, biomass allocation to each organ of seedling was well coordinated in different positions, which could lead to successful seedling recruitment.

### 4.2 Effects of seed position on trade-off strategies of seedling nutrient contents

Plant N and P contents and N:P ratios reflect the dynamic balance of nutrients and adaptation strategies of plants as affected by resource supply and demand (Koerselman and Meuleman, 1996; Jing et al., 2017), and are also closely associated with plant photosynthesis ability (de Groot et al., 2003). Generally, plants avoid damage in harsh environmental conditions and increase survival by maximizing organ strength (Shen et al., 2019b). In this study, emerged seedlings from seeds positioned beneath the deep litter layer generally had higher N and P contents than seedlings from other seed positions. This general pattern is consistent with the observations made by Donath and Eckstein (2011) and might be attributed to the strong positive correlation between litter thickness and light extinction (Sayer et al., 2006). With increasing litter thickness, light extinction directly offsets the photosynthetically active radiation received by plants (Xia et al., 2019). Emerged seedlings from seeds positioned beneath the deep litter layer increased investment in N and P contents to intercept light resources, and increase photosynthetic rates (Poorter et al., 2019). Nutrient contents are also transferred to roots to break through the litter mechanical barrier (Zhu et al., 2022b). Furthermore, the N and P contents of seedlings from seeds positioned on the forest floor were higher overall than that of seedlings from other seed positions. The likely cause of this result was that seedlings allocate more growth to root tissue to capture the most available resources (e.g., water or organic matter), thus achieving growth (Loydi et al., 2015; Yang et al., 2018). On the forest floor, water limitation decreases the adsorption and dissolution of inorganic P and blocks the upward transport of N and P because xylem embolisms form (He and Dijkstra, 2015; Yang, 2018). In this study, seedlings from seeds positioned on the forest floor had higher N and P contents in roots than in stems and leaves, which further confirmed the increased investment in root tissue. However, a moderate litter layer (~40 g of litter) overall decreased N and P contents of the shoot and root tissues compared with those of emerged seedlings from seeds positioned on the forest floor. Litter was assumed to alter the forest microenvironment (Donath and Eckstein, 2011) and minimize strong light radiation. In a moderate litter layer, litter reduces the evaporation rate of soil moisture and provides a shaded environment (Poorter et al., 2019), which further promotes seedling recruitment. In this case, seedlings accelerate the absorption and use of nutrient resources rather than balance supplies and demand to achieve rapid growth, which resulted in nutrient contents that were lower than those of other positions.

The N:P ratio is used to evaluate nutrient-limiting conditions in plants, and N:P ratio < 14, 14 < N:P ratios < 16, and N:P ratios >16 generally indicate limitations of N, both N and P, and P, respectively (Koerselman and Meuleman, 1996; Reich and Oleksyn, 2004; Han et al., 2005). In this study, N:P ratios (<14) were highest in leaves, intermediate in stems, and lowest in roots, which indicated that the growth of emerged seedlings was mainly limited by N (**Figure 4**). Leaf tissue synthesizes proteins via photosynthesis, and N-restricted seedlings obtain the most limited resource by increasing investment in leaves at the expense of non-photosynthetic tissues (Umaña et al., 2020). This trade-off directly leads to higher N:P ratios of leaves than of roots and stems. In this study, N:P ratios in each organ under a moderate litter layer were higher than those of other positions. The result indicated that the environment of the position was suitable for seedling growth because N and P contents were regulated, which can ultimately improve seedling survival (Han et al., 2005; Yang, 2018). The difference in position was likely because nutrients released by litter stimulated soil microbial activities through plant–soil feedback (Zhang et al., 2019; Wang et al., 2021a; Yang et al., 2021). When litter thickness exceeds the ability of a plant to adapt, physical effects caused by a deep litter layer may be a key factor inhibiting plant growth (Wellstein, 2015). Therefore, in a moderate litter layer, emerged seedlings alleviated nutrient limitations by altering N:P ratios (<14), which could directly influence seedling recruitment in subtropical forests.

### 4.3 Effects of seed position on seedling nutrient accumulation and use efficiency

Nutrient accumulation and use efficiency are important factors affecting plant regeneration and adaptation strategies (Han et al., 2005). A moderate litter layer (~40 g of litter) promoted N and P accumulation and nutrient use efficiencies in emerged seedlings (**Figure 5**). Such effects are likely associated with litter nutrient turnover rates and release (Berg, 2000). Rapidly decomposing litter increases soil nutrient availability for plant growth (Berg, 2000; Xu et al., 2013), thereby easing nutrient limitations for emerged seedlings. When decomposed slowly, litter maintains soil moisture and regulates soil temperature and relative humidity (Xu et al., 2013). This study concludes that the litter layer increased the diffusion of N and P in soil and thereby increased nutrient absorption and utilization by seedlings (Ellsworth et al., 2004). Thus, a moderate litter layer can provide an optimal growth environment for emerged seedlings in which roots assimilate and then shift N and P to other tissues (Boonman et al., 2020). In a moderate litter layer, seedlings can allocate growth to other tissues to compensate for the adverse effects of nutrient limitations by increasing nutrient accumulation and use efficiency and maintaining a nutrient balance (Yang et al., 2018). However, nutrient accumulation and use efficiency of emerged seedlings from seeds positioned beneath the deep litter layer were lower than those of seedlings in other positions. This result was likely caused by low amounts of nutrients released from litter (chemical effects) combined with mechanical barriers (physical effects). Physical effects are important factors affecting seedling emergence (Xu et al., 2013; Wellstein, 2015). When soil nutrients released by decomposing litter counteract the inhibition of physical barriers, seedlings from seeds positioned beneath a deep litter layer allocate additional growth to roots to expand the root area to absorb soil nutrients. Beneath a deep litter layer, plants can penetrate through litter cover to emerge and improve competitiveness (de Groot et al., 2003; Quested and Eriksson, 2006). Overall, seed relative positions influence the adaptability of emerged seedlings by altering nutrient accumulation and nutrient use efficiency of each organ.

### 4.4 Implications of trade-off strategies for regeneration in subtropical forests

In this study, relationships were explored between different positions of seeds and biomass and nutrient contents of seedlings of a dominant tree species to better understand how seed position affects regeneration in subtropical natural forests (**Figures 6**, **S2**). According to cluster analysis, biomass and nutrient allocation of emerged seedlings from seeds positioned above litter layers of different thicknesses, and those from seeds positioned beneath the deep litter layer and on the forest floor were clustered in one group. These results indicated that the variations of traits (biomass and nutrient) allocation were highly similar (Zhu et al., 2022b). Thus, emerged seedlings from these positions may increase the allocation to roots to obtain soil organic matter and water resources to adapt to an unfavorable environment, thus achieving rapid growth (Shen et al., 2019b). With continuous input, litter can regulate the physical and chemical environment of seed positions, and the amount of litter may be a critical factor in seedling regeneration (Wellstein, 2015). Similarly, to eliminate the negative effects caused by litter, emerged seedlings from seeds positioned beneath the deep litter layer expanded root area or increased leaf N contents to access limited resources and promote seedling growth. Comprehensive scores indicated that moderate litter cover (~40 g of litter) promoted the most seedling growth (**Figure 7**), and thus, positioning seeds in a moderately thick litter layer might be the best approach to regulate biomass and nutrient allocation of emerged seedlings in subtropical forests.

**Figure 8:**
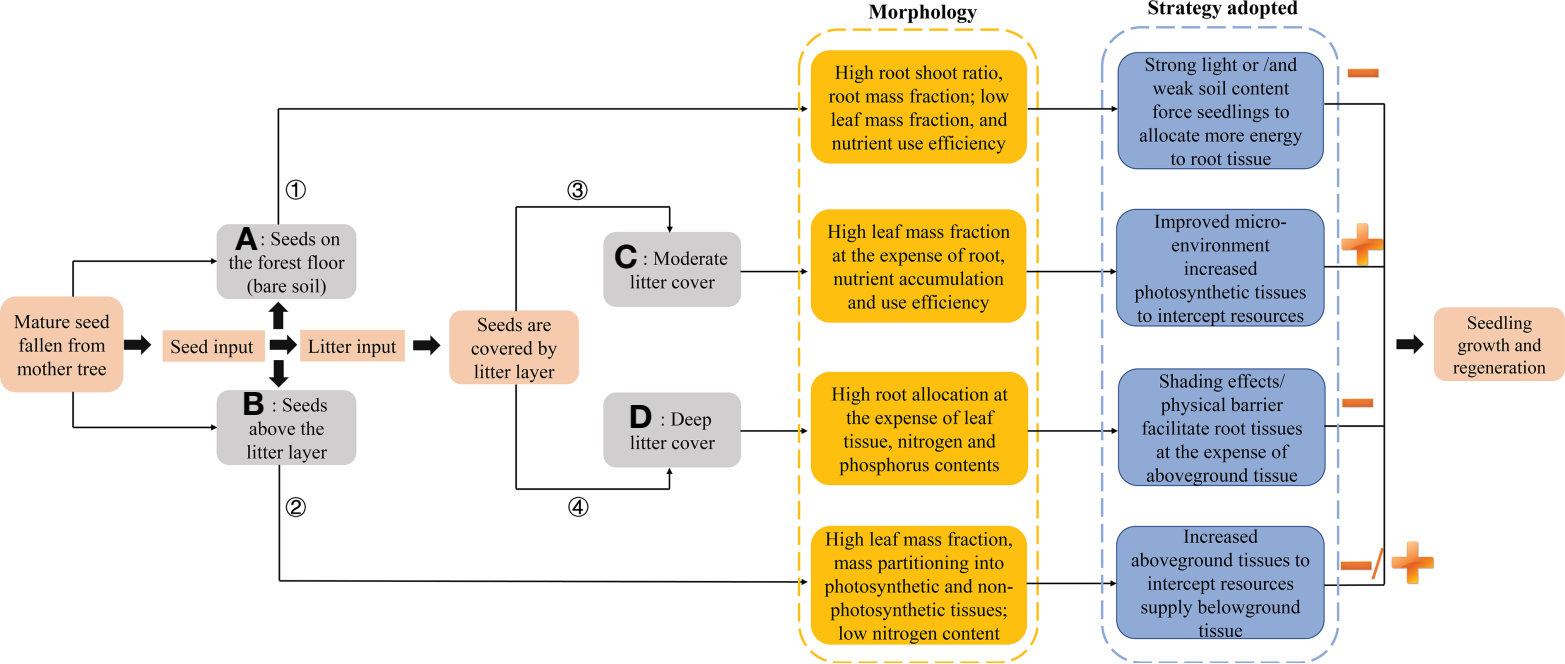
Trade-offs in seedling adaptability depend on seed positions. (1) Gray box represents seed positions; orange and blue boxes indicate morphological characteristics of emerged seedlings from seeds positioned on the forest floor and litter layer, and their adopted resource strategies, respectively. Addition (+) and subtraction **(-)** signs represent positive and negative effects, respectively, on seedling growth from seeds in different positions, whereas -/+ indicates first promotion and then inhibition of seedling growth in a position. (2) Main results: when mature seeds fall from mother trees, they may be positioned **(A)** on the forest floor (bare soil) or **(B)** above the litter layer. Seedlings from seeds positioned on the forest floor allocated growth to roots to achieve growth. With litter input, seedlings from seeds positioned in deep litter **(D)**/top of litter had relatively high leaf mass fraction at the expense of root investment. Moderate litter layer cover **(C)** (~40 g of litter) increased allocation to photosynthetic tissue at the expense of root tissue to promote the natural regeneration of seedlings in forests.

Root, stem, and leaf N:P ratios <14, as well as relatively high leaf N:P ratios and relatively low coefficients of variation (**Figure S4**), further confirmed the hypothesis that nutrient limitations for plant growth are best evaluated by leaf N:P ratios, compared with those of other tissues (Güsewell, 2004; He et al., 2008). A comprehensive evaluation of biomass and nutrient traits in seedlings from different seed positions demonstrated that the entropy weight vectors of root N:P ratios and P use efficiency were higher than those of other traits. The results further verified that P is the main factor affecting the rapid growth of plants during regeneration in subtropical evergreen broad-leaved forests (Han et al., 2005). It was also hypothesized that litter regulates the physical and chemical environment of seedlings during recruitment and that interactions between such effects may produce conflicting responses between above- and belowground biomass allocation (**Figure 8**). Thus, studying the biomass and nutrient allocation of seedlings from seeds positioned above, within, or beneath the forest floor and litter layer is a powerful approach to understanding forest regeneration. However, other variables that affect the allocation strategies of seedlings, such as the soil environment and differences between lab and field experiments (He et al., 2020), were not tested in this study, although such variables may also be important in regulating seedling regeneration and thus deserve further study. Likewise, allocation strategies of seedlings in different age classes or habitats should be considered in future research. Finally, long-term responses in subtropical forests also need to be monitored.

## 5 Conclusion

To investigate factors affecting seedling regeneration in subtropical forests, this study explored the effects of different seed positions on above- and belowground biomass and nutrient contents of emerged seedlings of a dominant tree species. One critical finding was that biomass allocation in different organs was well coordinated. Emerged seedlings from seeds positioned above litter and beneath a moderate litter layer (~40 and 80 g of litter) had higher leaf allocation at the expense of root and stem tissues than seedlings from other positions. Emerged seedlings from seeds positioned beneath a deep litter layer (~120 and 160 g of litter) had higher root allocation at the expense of leaf tissue than seedlings from other positions, which was necessary to break through the physical barrier of litter. Biomass and nutrient allocation of emerged seedlings from seeds positioned on the forest floor indicated a decrease in investment in shoot tissue to achieve growth. Traits of biomass and nutrient were also comprehensively analyzed, and the analysis indicated that seedlings from seeds positioned beneath a moderate litter layer (~40 g of litter) facilitated the regeneration of a dominant tree species in the natural community, because those seedlings had relatively high N and P accumulation and nutrient use efficiency (N:P ratios < 14). Thus, protective mechanisms that occur in seeds positioned beneath a litter layer are particularly important for the natural regeneration of seedlings. In the future, seedling regeneration in subtropical natural forests could be promoted by adjusting litter thickness or adding N elements.

## Data availability statement

The raw data supporting the conclusions of this article will be made available by the authors, without undue reservation.

## Author contributions

JZ: conceptualization, formal analysis, investigation, methodology, writing–original draft, writing–review, and editing. LJ: investigation and formal analysis. LC: writing–review and editing. XJ: investigation. CX: investigation. JL: conceptualization, supervision, writing–review, editing, and funding acquisition. YY: writing–review and editing. ZH: conceptualization, supervision, writing–review, editing, and funding acquisition. All authors contributed to the article and approved the submitted version.

## References

[B1] Berg B. (2000). Litter decomposition and organic matter turnover in northern forest soils. For. Ecol. Manage. 133, 13–22. doi: 10.1016/S0378-1127(99)00294-7

[B2] Bhadouria R. Srivastava P. Singh R. Tripathi S. Singh H. Raghubanshi A. S. (2017). Tree seedling establishment in dry tropics: an urgent need of interaction studies. Environ. Syst. Decis. 37, 88–100. doi: 10.1007/s10669-017-9625-x

[B3] Bhadouria R. Srivastava P. Singh R. Tripathi S. Verma P. Raghubanshi A. S. (2020). Effects of grass competition on tree seedlings growth under different light and nutrient availability conditions in tropical dry forests in India. Ecol. Res. 35, 807–818. doi: 10.1111/1440-1703.12131

[B4] Boonman C. C. F. van Langevelde F. Oliveras I. Couédon J. Luijken N. Martini D.. (2020). On the importance of root traits in seedlings of tropical tree species. New Phytol. 227, 156–167. doi: 10.1111/nph.16370 31834943 PMC7317509

[B5] Bridgham S. D. Pastor J. (1995). Nutrient-use efficiency: a litterfall index, a model, and a test along a nutrient availability. Am. Nat. 145, 1–21. doi: 10.1086/285725

[B6] de Groot C. C. van den Boogaard R. Marcelis L. F. M. Harbinson J. Lambers H. (2003). Contrasting effects of n and p deprivation on the regulation of photosynthesis in tomato plants in relation to feedback limitation. J. Exp. Bot. 54, 1957–1967. doi: 10.1093/jxb/erg193 12837813

[B7] de Mendiburu F. (2009). Una herramienta de analisis estadistico para la investigacion agricola. master’s thesis (Rímac, Peru: Universidad Nacional de Ingenieria (UNI-PERU).

[B8] Donath T. W. Eckstein R. L. (2011). Litter effects on seedling establishment interact with seed position and earthworm activity. Plant Biol. 14, 163–170. doi: 10.1111/j.1438-8677.2011.00490.x 21972886

[B9] Du X. J. Guo Q. F. Gao X. M. Ma K. P. (2007). Seed rain, soil seed bank, seed loss and regeneration of *Castanopsis fargesii* (Fagaceae) in a subtropical evergreen broad-leaved forest. For. Ecol. Manage. 238, 212–219. doi: 10.1016/j.foreco.2006.10.018

[B10] Ellsworth J. W. Harrington R. A. Fownes J. H. (2004). Seedling emergence, growth, and allocation of oriental bittersweet: effects of seed input, seed bank, and forest floor litter. For. Ecol. Manage. 190, 255–264. doi: 10.1016/j.foreco.2003.10.015

[B11] Friendly M. (2002). Corrgrams: exploratory displays for correlation matrices. Am. Stat. 56, 316–324. doi: 10.1198/000313002533

[B12] Güsewell S. (2004). N:P ratios in terrestrial plants: variation and functional significance. New Phytol. 164, 243–266. doi: 10.1111/j.1469-8137.2004.01192.x 33873556

[B13] Han W. X. Fang J. Y. Guo D. L. Zhang Y. (2005). Leaf nitrogen and phosphorus stoichiometry across 753 terrestrial plant species in China. New Phytol. 168, 377–385. doi: 10.1111/j.1469-8137.2005.01530.x 16219077

[B15] He M. Z. Dijkstra F. A. (2015). Drought effect on plant nitrogen and phosphorus: a meta-analysis. New Phytol. 204, 924–931. doi: 10.1111/nph.12952 25130263

[B16] He Z. S. Wang L.J. Jiang L. Wang Z. Liu J.F. Xu D.W.. (2019). Effect of microenvironment on species distribution patterns in the regeneration layer of forest gaps and non-gaps in a subtropical natural forest, China. Forests 10, 90. doi: 10.3390/f10020090

[B17] He Z. S. Tang R. Li M. J. Jin M. R. Xin C. Liu J. F.. (2020). Response of photosynthesis and chlorophyll fluorescence parameters of *Castanopsis kawakamii* seedlings to forest gaps. Forests 11, 21. doi: 10.3390/f11010021

[B14] He J. S. Wang L. Flynn D. F. B. Wang X. P. Ma W. H. Fang J. Y. (2008). Leaf nitrogen: phosphorus stoichiometry across Chinese grassland biomes. Oecologia 155, 301–310. doi: 10.1007/s00442-007-0912-y 18278518

[B18] Hodáňová D. (1981). Plant strategies and vegetation processes. Biol. Plant 23, 254. doi: 10.1007/BF02895358

[B19] Huang L. Jin C. Zhou L. H. Song K. Qian S. H. Lin D. M.. (2021). Benefit vs. cost trade-offs of masting across seed-to-seedling transition for a dominant subtropical forest species. J. Ecol. 109, 3087–3098. doi: 10.1111/1365-2745.13722

[B20] Huang L. Zhou L. H. Wang J. M. Jin C. Hu S. W. Qian S. H.. (2020). Short-term decline of *Castanopsis fargesii* adult trees promotes conspecific seedling regeneration: the complete process from seed production to seedling establishment. Ecol. Evol. 10, 10657–10671. doi: 10.1002/ece3.6719 33072287 PMC7548161

[B21] Jing H. Zhou H. X. Wang G. L. Xue S. Liu G. B. Duan M. C. (2017). Nitrogen addition changes the stoichiometry and growth rate of different organs in *Pinus tabuliformis* seedlings. Front. Plant Sci. 8. doi: 10.3389/fpls.2017.01922 PMC5681934 29163630

[B22] Kassambara A. Mundt F. (2017) Factoextra: extract and visualize the results of multivariate data analyses. retrieved from. Available at: http://www.sthda.com/english/rpkgs/factoextra.

[B23] Koerselman W. Meuleman A. F. M. (1996). The vegetation N:P ratio: a new tool to detect the nature of nutrient limitation. J. Appl. Ecol. 33, 1441–1450. doi: 10.2307/2404783

[B24] Ledo A. Paul K. I. Burslem D. F. R. P. Ewel J. J. Barton C. Battaglia M.. (2018). Tree size and climatic water deficit control root to shoot ratio in individual trees globally. New Phytol. 217, 8–11. doi: 10.1111/nph.14863 29058312

[B25] Liang M. X. Liu X. B. Parker I. M. Johnson D. Zheng Y. Luo S.. (2019). Soil microbes drive phylogenetic diversity-productivity relationships in a subtropical forest. Sci. Adv. 5, eaax5088. doi: 10.1126/sciadv.aax5088 31681847 PMC6810308

[B26] Loydi A. Donath T. W. Otte A. Eckstein R. L. (2015). Negative and positive interactions among plants: effects of competitors and litter on seedling emergence and growth of forest and grassland species. Plant Biol. 17, 667–675. doi: 10.1111/plb.12287 25381837

[B27] Luo Y. J. Wang X. K. Ouyang Z. Y. Lu F. Feng L. G. Tao J. (2020). A review of biomass equations for china’s tree species. Earth Syst. Sci. Data 12, 21–40. doi: 10.5194/essd-12-21-2020

[B28] Ma H. Z. Mo L. D. Crowther T. W. Maynard D. S. van den Hoogen J. Stocker B. D.. (2021). The global distribution and environmental drivers of aboveground versus belowground plant biomass. Nat. Ecol. Evol. 5, 1110–1122. doi: 10.1038/s41559-021-01485-1 34168336

[B29] Nakagawa S. Cuthill I. C. (2007). Effect size, confidence interval and statistical significance: a practical guide for biologists. Biol. Rev. 82, 591–605. doi: 10.1111/j.1469-185X.2007.00027.x 17944619

[B30] Nuñez N. H. Chazdon R. L. Russo S. E. (2021). Seed rain–successional feedbacks in wet tropical forests. Ecology 102, e03362. doi: 10.1002/ecy.3362 33834498

[B31] Ottaviani G. Molina-Venegas R. Charles-Dominique T. Chelli S. Campetella G. Canullo R.. (2020). The neglected belowground dimension of plant dominance. Trends Ecol. Evol. 35, 763–766. doi: 10.1016/j.tree.2020.06.006 32650986

[B32] Perea A. J. Wiegand T. Garrido J. L. Rey P. J. Alcántara J. M. (2021). Legacy effects of seed dispersal mechanisms shape the spatial interaction network of plant species in Mediterranean forests. J. Ecol. 109, 3670–3684. doi: 10.1111/1365-2745.13744

[B33] Poorter H. Ülo N. Ntagkas N. Siebenkäs A. Mäenpää M. Matsubara S.. (2019). A meta-analysis of plant responses to light intensity for 70 traits ranging from molecules to whole plant performance. New Phytol. 223, 1073–1105. doi: 10.1111/nph.15754 30802971

[B34] Quested H. Eriksson Q. (2006). Litter species composition influences the performance of seedlings of grassland herbs. Funct. Ecol. 20, 522–532. doi: 10.1111/j.1365-2435.2006.01131.x

[B35] R Delopment Core Team (2020). R: a language and environment for statistical computing (Vienna, Austria: R Foundation for Statistical Computing). Available at: http://www.R-project.org/.

[B36] Reich P. B. Oleksyn J. (2004). Global patterns of plant leaf n and p in relation to temperature and latitude. Proc. Natl. Acad. Sci. U.S.A. 101, 11001–11006. doi: 10.1073/pnas.0403588101 15213326 PMC503733

[B37] Rotundo J. L. Aguiar M. R. (2005). Litter effects on plant regeneration in arid lands: a complex balance between seed retention, seed longevity and soil-seed contact. J. Ecol. 93, 829–838. doi: 10.1111/j.1365-2745.2005.01022.x

[B39] Ruprecht E. Józsa J. Ölvedi T. B. Simon J. (2010). Differential effects of several “litter” types on the germination of dry grassland species. J. Veg. Sci. 21, 1069–1081. doi: 10.1111/j.1654-1103.2010.01206.x

[B38] Ruprecht E. Szabó A. (2012). Grass litter is a natural seed trap in long-term undisturbed grassland. J. Veg. Sci. 23, 495–504. doi: 10.1111/j.1654-1103.2011.01376.x

[B40] Sayer E. J. Tanner E. V. J. Cheesman A. W. (2006). Increased litterfall changes fine root distribution in a moist tropical forest. Plant Soil 281, 5–13. doi: 10.1007/s11104-005-6334-x

[B41] Shen Y. Gilbert G. S. Li W. B. Fang M. Lu H. P. Yu S. X. (2019a). Linking aboveground traits to root traits and local environment: implications of the plant economics spectrum. Front. Plant Sci. 10. doi: 10.3389/fpls.2019.01412 PMC6831723 31737024

[B42] Shen Y. Umaña M. N. Li W. B. Fang M. Chen Y. X. Lu H. P.. (2019b). Coordination of leaf, stem and root traits in determining seedling mortality in a subtropical forest. For. Ecol. Manage. 446, 285–292. doi: 10.1016/j.foreco.2019.05.032

[B43] Umaña M. N. Arellano G. Swenson N. G. Zambrano J. (2021). Tree seedling trait optimization and growth in response to local-scale soil and light variability. Ecology 102, e03252. doi: 10.1002/ecy.3252 33219522

[B44] Umaña M. N. Cao M. Lin L. X. Swenson N. G. Zhang C. C. (2020). Trade-offs in above and belowground biomass allocation influencing seedling growth in a tropical forest. J. Ecol. 109, 1184–1193. doi: 10.1111/1365-2745.13543

[B45] Umaña M. N. Forero-Montaña J. Muscarella R. Nytch C. J. Thompson J. Uriarte M.. (2016). Interspecific functional convergence and divergence and intraspecific negative density dependence underlie the seed-to-seedling transition in tropical trees. Am. Nat. 187, 99–109. doi: 10.1086/684174 27277406

[B46] Walck J. L. Hidayati S. N. Dixon K. W. Thompson K. Poschlod P. (2011). Climate change and plant regeneration from seed. Glob. Change Biol. 17, 2145–2161. doi: 10.1111/j.1365-2486.2010.02368.x

[B48] Wang X. L. Liu J. F. He Z. S. Xing C. Zhu J. Gu X. G.. (2021b). Forest gaps mediate the structure and function of soil microbial community in a *Castanopsis kawakamii* forest. Ecol. Indic. 122, 107288. doi: 10.1016/j.ecolind.2020.107288

[B47] Wang J. N. Xu B. Wu Y. Gao J. Shi F. S. Wu N. (2021a). Effect of inflorescence litter from distinct species and life forms on soil nutrients and microbial biomass in the eastern Tibetan plateau. Glob. Ecol. Conserv. 31, e01825. doi: 10.1016/j.gecco.2021.e01825

[B49] Weemstra M. Zambrano J. Allen D. Umaña M. N. (2021). Tree growth increases through opposing above and belowground resource strategies. J. Ecol. 109, 3502–3512. doi: 10.1111/1365-2745.13729

[B50] Wellstein C. (2015). Seed-litter-position drives seedling establishment in grassland species under recurrent drought. Plant Biol. 14, 1006–1010. doi: 10.1111/j.1438-8677.2012.00635.x 22822918

[B51] Werden L. K. Holl K. D. Rosales J. A. Sylvester J. M. Zahawi R. A. (2020). Effects of dispersal-and niche-based factors on tree recruitment in tropical wet forest restoration. Ecol. Appl. 30, e02139. doi: 10.1002/eap.2139 32335980

[B52] Xia L. Song X. Y. Fu N. Cui S. Y. Li L. J. Li H. Y.. (2019). Effects of forest litter cover on hydrological response of hillslopes in the loess plateau of China. Catena 181, 104076. doi: 10.1016/j.catena.2019.104076

[B53] Xu S. Liu L. L. Sayer E. J. (2013). Variability of above-ground litter inputs alters soil physicochemical and biological processes: a meta-analysis of litterfall-manipulation experiments. Biogeosciences 10, 7423–7433. doi: 10.5194/bg-10-7423-2013

[B54] Yang H. (2018). Effects of nitrogen and phosphorus addition on leaf nutrient characteristics in a subtropical forest. Trees 32, 383–391. doi: 10.1007/s00468-017-1636-1

[B57] Yang Y. C. Huang L. Qian S. H. Fukuda K. (2015). Completing the life history of *Castanopsis fargesii*: changes in the seed dispersal, seedling and sapling recruitment patterns. Eur. J. For. Res. 134, 1143–1154. doi: 10.1007/s10342-015-0916-9

[B56] Yang Y. Liu B. R. An S. S. (2018). Ecological stoichiometry in leaves, roots, litters and soil among different plant communities in a desertified region of northern China. Catena 166, 328–338. doi: 10.1016/j.catena.2018.04.018

[B55] Yang X. L. Wang X. T. Xiao S. Liu Z. Y. Zhou X. H. Du G. Z.. (2021). Dominant plants affect litter decomposition mainly through modifications of the soil microbial community. Soil Biol. Biochem. 161, 108399. doi: 10.1016/j.soilbio.2021.108399

[B59] Zhang P. Li B. Wu J. H. Hu S. J. (2019). Invasive plants differentially affect soil biota through litter and rhizosphere pathways: a meta-analysis. Ecol. Lett. 22, 200–210. doi: 10.1111/ele.13181 30460738

[B60] Zhang X. Y. Ni X. Y. Heděnec P. Yue K. Wei X. Y. Yang J.. (2022). Litter facilitates plant development but restricts seedling establishment during vegetation regeneration. Funct. Ecol. 36, 3134–3147. doi: 10.1111/1365-2435.14200

[B58] Zhang C. H. Xi N. X. (2021). Precipitation changes regulate plant and soil microbial biomass *via* plasticity in plant biomass allocation in grasslands: a meta-analysis. Front. Plant Sci. 12, 614968. doi: 10.3389/fpls.2021.614968 33719286 PMC7947227

[B61] Zhong C. H. Yang Q. C. Liang J. Ma H. Y. (2021). Fuzzy comprehensive evaluation with AHP and entropy methods and health risk assessment of groundwater in yinchuan basin, northwest China. Environ. Res. 204, 111956. doi: 10.1016/j.envres.2021.111956 34454937

[B62] Zhu J. Jiang L. Zhu D. H. Xing C. Jin M. R. Liu J. F.. (2022a). Forest gaps regulate seed germination rate and radicle growth of an endangered plant species in a subtropical natural forest. Plant Diversity 44, 445–454. doi: 10.1016/j.pld.2021.10.003 36187548 PMC9512644

[B63] Zhu J. Liu J. F. Xing C. Jiang L. Wang X. L. He Z. S.. (2022b). Effect of seeds dispersal position on the root morphology and growth characteristics of *Castanopsis kawakamii* seedlings. Acta Ecol. Sin. 42, 4065–4075. doi: 10.5846/stxb202102060385

[B64] Zou Z. H. Yi Y. Sun J. N. (2006). Entropy method for determination of weight of evaluating indicators in fuzzy synthetic evaluation for water quality assessment. J. Environ. Sci. 18, 1020–1023. doi: 10.1016/S1001-0742(06)60032-6 17278765

