# Supplementary material for: Tree seedling growth allocation of Castanopsis kawakamii is determined by seed-relative positions
Source: Front Plant Sci. 2023 Jun 2;14:1099139. doi: 10.3389/fpls.2023.1099139 (PMC10272420; doi:10.3389/fpls.2023.1099139)
Supplement: Supplementary file 1 [file DataSheet_1.docx]

***Supplementary Materials***

| 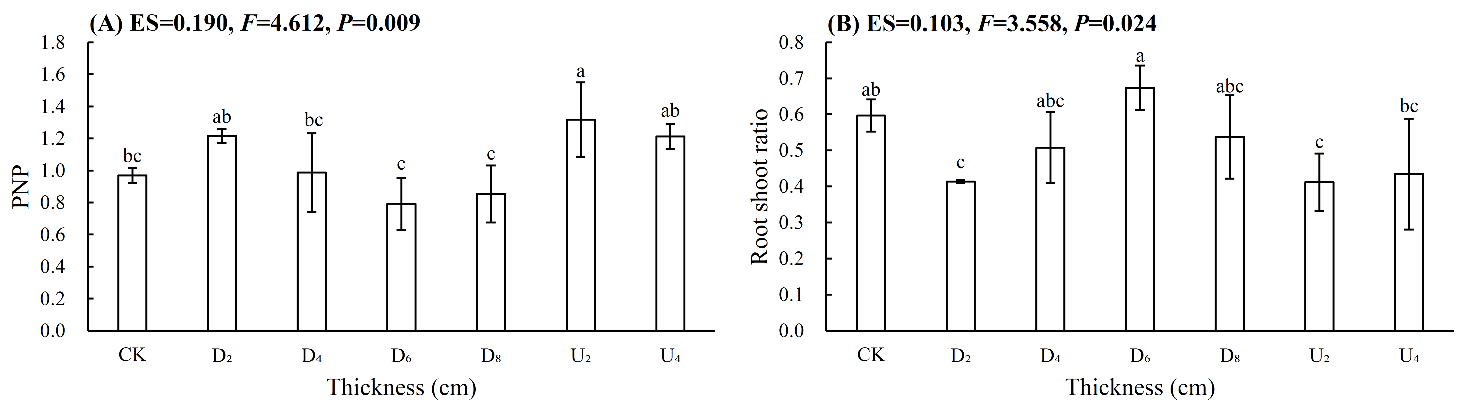 |
| --- |
| **Supplementary Figure 1.** The (A) mass partitioning into photosynthetic and non-photosynthetic tissues (PNP) and (B) root shoot ratio of *Castanopsis kawakamii* seedling. CK represents emerged seedlings from seeds positioned on the forest floor (bare soil); D_2_, D_4_, D_6_, and D_8_ represent emerged seedlings from seeds positioned beneath the litter layer with cover of 2 cm, 4 cm, 6 cm, and 8 cm of litter, respectively; U_2_ and U_4_ represent emerged seedlings from seeds positioned above the litter 2 cm and 4 cm thick, respectively. Different lowercase letters (a, b) indicate significant differences (*P*<0.05), ES indicate the effect size; *P* indicates probability value; *F* indicates *F* test value; error bars represent one standard deviation; the same below (df = 6). |
| **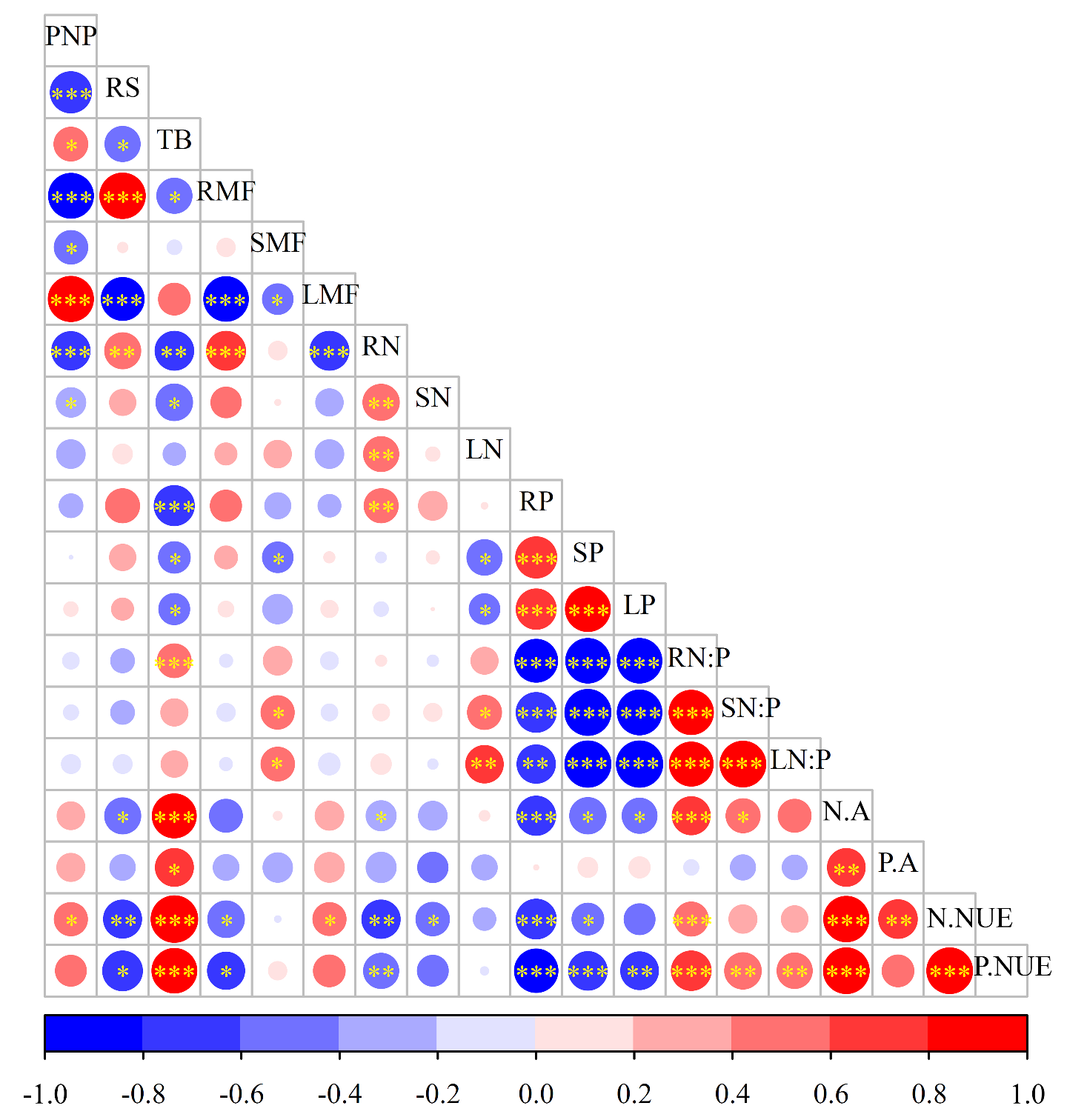** |
| **Supplementary Figure 2.** The correlation analysis between biomass and nutrient contents of *Castanopsis kawakamii* seedlings. PNP and RS represent mass partitioning into photosynthetic and non-photosynthetic tissues, root shoot ratio. TB, RMF, SMF and LMF indicate total plant biomass, root mass fraction, stem mass fraction, leaf mass fraction, respectively. RN, SN, and LN represent root, stem and leaf nitrogen contents, respectively. RP, SP, and LP indicate root, stem and leaf phosphorus contents, respectively. RN:P, SN:P, and LN:P represent total nitrogen contents divided by total phosphorus of root, stem and leaf, respectively. NA, PA, N.NUE, and P.NUE indicate nitrogen accumulation, phosphorus accumulation, nitrogen nutrient use efficiency, phosphorus nutrient use efficiency, respectively; The same below. *, **, *** represent significant level at 0.05, 0.01, 0.001, respectively. |
| **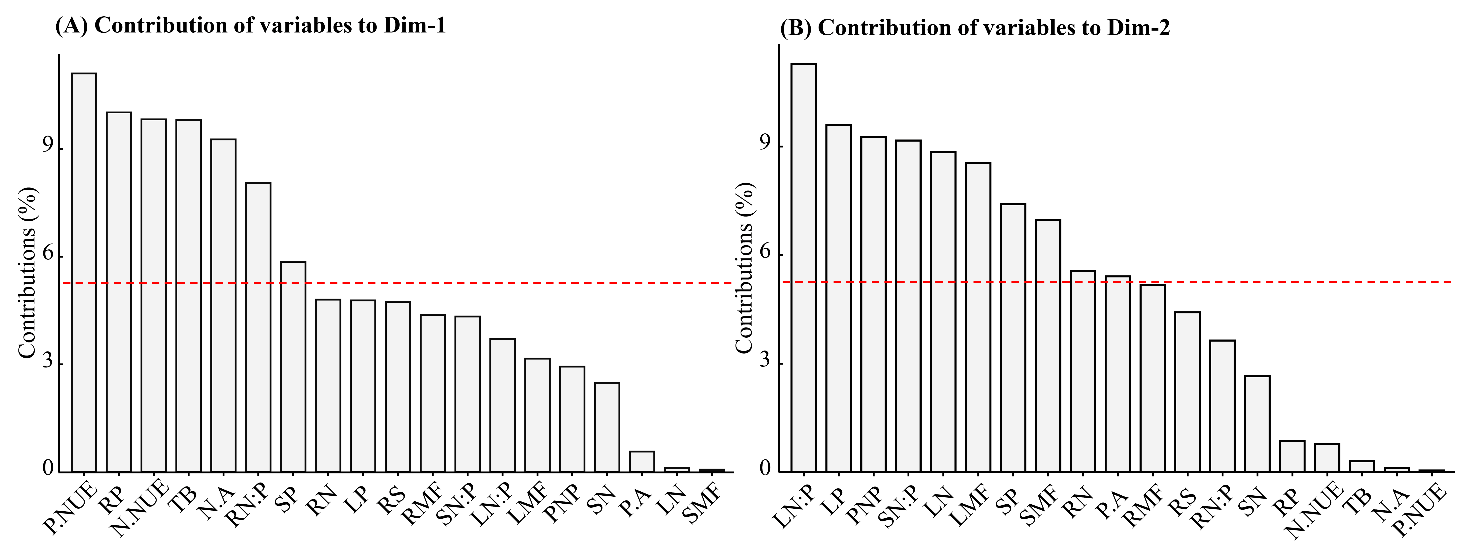** |
| **Supplementary Figure 3.** Contribution of variables to Dimension 1 (A) and Dimension 2 (B). Red dotted line represents the average contribution rate of each variable. |

**
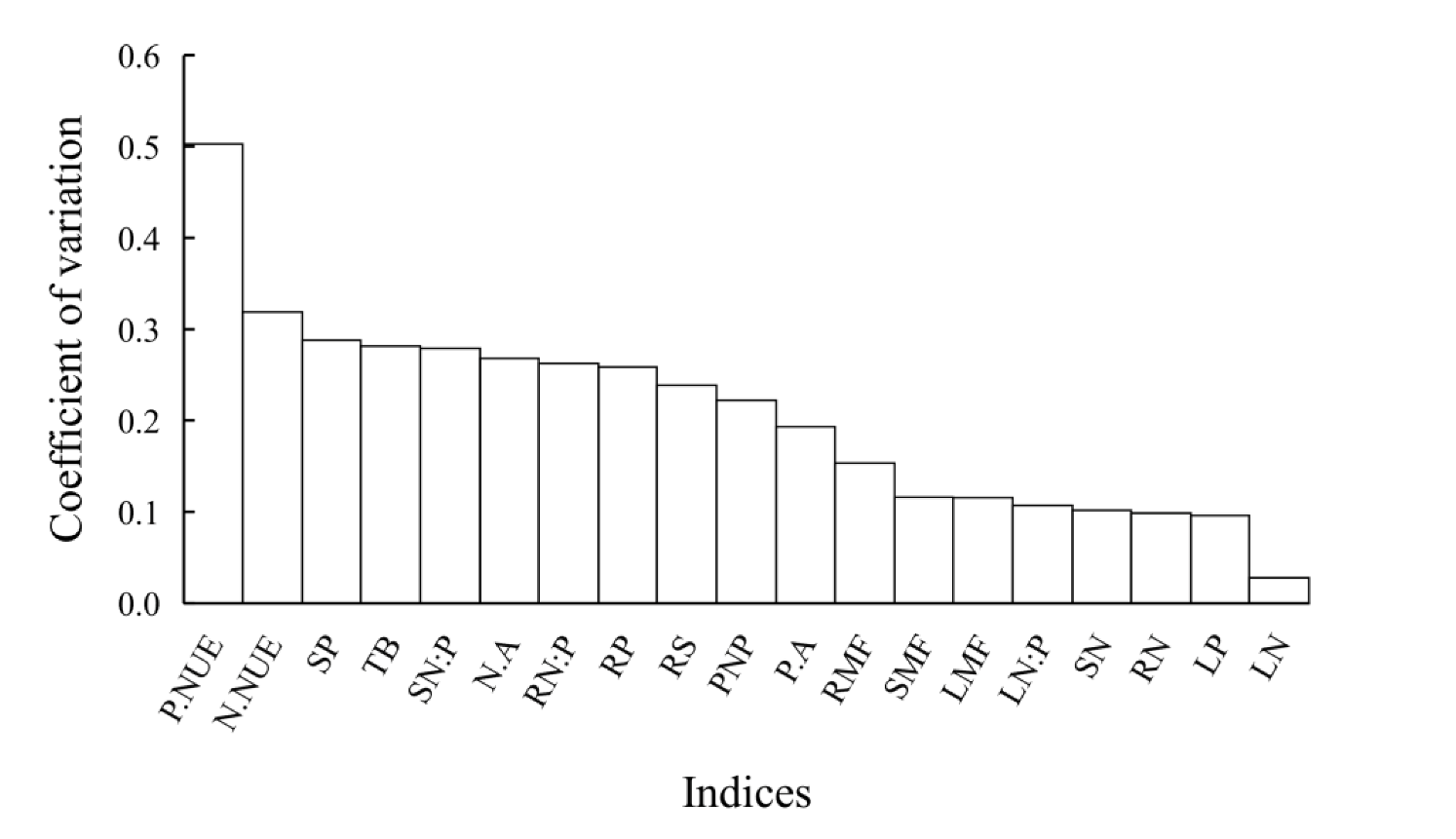
**

**Supplementary Figure 4.** Coefficients of variation of biomass and nutrient indices of seedling.

**Supplementary Table S1.** Relationships of within traits indices of *Castanopsis kawakamii* seedlings

| Traits | 95% Confidence interval | | | | |
| --- | --- | --- | --- | --- | --- |
|  | Lower limit | Upper limit |  | Lower limit | Upper limit |
| PNP | 0.943 | 1.155 | SP | 2.077 | 2.704 |
| RS | 0.455 | 0.567 | LP | 1.940 | 2.117 |
| TB | 1.118 | 1.446 | RN:P | 3.230 | 4.106 |
| RMF | 30.874 | 35.513 | SN:P | 4.822 | 6.225 |
| SMF | 15.732 | 17.486 | LN:P | 8.113 | 8.944 |
| LMF | 47.556 | 52.840 | N. A | 18.071 | 23.086 |
| RN | 15.762 | 17.246 | P. A | 1.345 | 6.669 |
| SN | 11.707 | 12.840 | N.NUE | 0.068 | 0.092 |
| LN | 16.916 | 17.350 | P.NUE | 0.361 | 0.575 |
| RP | 4.224 | 5.349 | CA | 0.047 | 0.049 |

Notes: PNP and RS represent mass partitioning into photosynthetic and non-photosynthetic tissues, root shoot ratio. TB, RMF, SMF and LMF indicate total plant biomass, root mass fraction, stem mass fraction, leaf mass fraction, respectively. RN, SN, and LN represent root, stem and leaf nitrogen contents, respectively. RP, SP, and LP indicate root, stem and leaf phosphorus contents, respectively. RN:P, SN:P, and LN:P represent total nitrogen contents divided by total phosphorus of root, stem and leaf, respectively. NA, PA, N.NUE, and P.NUE indicate nitrogen accumulation, phosphorus accumulation, nitrogen nutrient use efficiency, phosphorus nutrient use efficiency, respectively. CA indicate the comprehensive score.

**Supplementary Table S2.** Evaluation weights of traits indices of *Castanopsis kawakamii* seedlings

| Traits | Indicators | Entropy weight vector |
| --- | --- | --- |
| Biomass traits | Mass partitioning into photosynthetic and non-photosynthetic tissues | 0.040 |
|  | Root shoot ratio | 0.039 |
|  | Total biomass | 0.053 |
|  | Root mass fraction | 0.033 |
|  | Stem mass fraction | 0.060 |
|  | Leaf mass fraction | 0.042 |
| Nutrient contents | Root nitrogen contents | 0.051 |
|  | Stem nitrogen contents | 0.044 |
|  | Leaf nitrogen contents | 0.049 |
|  | Root phosphorus contents | 0.065 |
|  | Stem phosphorus contents | 0.067 |
|  | Leaf phosphorus contents | 0.056 |
|  | Root N:P ratios | 0.078 |
|  | Stem N:P ratios | 0.048 |
|  | Leaf N:P ratios | 0.048 |
|  | Nitrogen accumulation | 0.059 |
|  | Phosphorus accumulation | 0.046 |
|  | Nitrogen nutrient use efficiency | 0.058 |
|  | Phosphorus nutrient use efficiency | 0.066 |
